# Epstein-Barr Virus Latency in B Cells Leads to Epigenetic Repression and CpG Methylation of the Tumour Suppressor Gene *Bim*

**DOI:** 10.1371/journal.ppat.1000492

**Published:** 2009-06-26

**Authors:** Kostas Paschos, Paul Smith, Emma Anderton, Jaap M. Middeldorp, Robert E. White, Martin J. Allday

**Affiliations:** 1 Department of Virology, Faculty of Medicine, Imperial College London, London, United Kingdom; 2 Breakthrough Breast Cancer Research Centre, The Institute of Cancer Research, London, United Kingdom; 3 Department of Pathology, VU University Medical Centre, Amsterdam, The Netherlands; Emory University, United States of America

## Abstract

In human B cells infected with Epstein-Barr virus (EBV), latency-associated virus gene products inhibit expression of the pro-apoptotic Bcl-2-family member Bim and enhance cell survival. This involves the activities of the EBV nuclear proteins EBNA3A and EBNA3C and appears to be predominantly directed at regulating Bim mRNA synthesis, although post-transcriptional regulation of Bim has been reported. Here we show that protein and RNA stability make little or no contribution to the EBV-associated repression of Bim in latently infected B cells. However, treatment of cells with inhibitors of histone deacetylase (HDAC) and DNA methyltransferase (DNMT) enzymes indicated that epigenetic mechanisms are involved in the down-regulation of *Bim*. This was initially confirmed by chromatin immunoprecipitation analysis of histone acetylation levels on the *Bim* promoter. Consistent with this, methylation-specific PCR (MSP) and bisulphite sequencing of regions within the large CpG island located at the 5′ end of *Bim* revealed significant methylation of CpG dinucleotides in all EBV-positive, but not EBV-negative B cells examined. Genomic DNA samples exhibiting methylation of the *Bim* promoter included extracts from a series of explanted EBV-positive Burkitt's lymphoma (BL) biopsies. Subsequent analyses of the histone modification H3K27-Me3 (trimethylation of histone H3 lysine 27) and CpG methylation at loci throughout the *Bim* promoter suggest that in EBV-positive B cells repression of *Bim* is initially associated with this repressive epigenetic histone mark gradually followed by DNA methylation at CpG dinucleotides. We conclude that latent EBV initiates a chain of events that leads to epigenetic repression of the tumour suppressor gene *Bim* in infected B cells and their progeny. This reprogramming of B cells could have important implications for our understanding of EBV persistence and the pathogenesis of EBV-associated disease, in particular BL.

## Author Summary

Bim is a cellular inducer of programmed cell death (pcd), so the level of Bim is a critical regulator of lymphocyte survival and reduced expression enhances lymphomagenesis in mice and humans. Regulation of *Bim* is uniquely important in the pathogenesis of Burkitt's lymphoma (BL), since in this human childhood cancer the *Myc* gene is deregulated by chromosomal translocation and Myc can induce pcd via Bim. Latent EBV represses Bim expression, and here we have discovered that this involves mechanisms that reprogramme B cells and their progeny. EBV does not significantly alter Bim protein or RNA stability, but relief of EBV-mediated repression by specific inhibitors suggested it involves modifications to chromatin. Consistent with this, reduced histone acetylation and increased levels of DNA methylation on the *Bim* promoter were found after latent EBV infection. Further analysis suggested that the DNA methylation is preceded by repression mediated via a polycomb protein repressive complex targeting the *Bim* gene. By initiating the heritable suppression of *Bim*, EBV increases the likelihood of B lymphomagenesis in general and BL in particular. This reprogramming of B cells by EBV may also play a role in the development of other chronic disorders such as autoimmune disease and suggests a general mechanism that could contribute to the pathogenesis associated with other microorganisms.

## Introduction

EBV is a B lymphotropic gammaherpes virus that asymptomatically and persistently infects >90% of humans; it occasionally causes infectious mononucleosis in adolescents and in rare instances is associated with the development of several different types of B cell lymphoma and various epithelial tumours [1]. EBV can also produce B cell lymphomas in non-human primates and induce the continuous proliferation (‘immortalisation’) of primary human B cells *in vitro*. The lymphoblastoid cell lines (LCLs) that are produced in culture carry the viral genome as extra-chromosomal episomes and express only nine ‘latent’ EBV proteins. There are six nuclear antigens (EBNAs 1, 2, 3A, 3B, 3C & LP) and three membrane associated proteins (LMP1, LMP2A & 2B); together with several RNA species, these factors activate the quiescent B cells from G0 into the cell cycle, initiate and sustain proliferation and maintain the viral episome in its extra-chromosomal state. Using recombinant viruses it has been shown that of all the transcripts expressed in LCLs only EBNA1, 2, 3A, 3C, -LP and LMP1 are essential for the efficient transformation of primary B cells into LCLs (reviewed in [2]).

Current data on the normal asymptomatic persistence of EBV in humans are consistent with the viral genome residing long-term in a resting memory B cell population. However, in order to establish persistence, EBV infects non-dividing naïve B cells and drives these to proliferate as activated B blasts that express all the viral proteins found in LCLs. The transient expansion of an infected B blast population is accompanied by their differentiation, probably in germinal centres, to become centroblasts and centrocytes and finally resting memory B cells. The precise series of events that the EBV-positive B cells undergo to reach the memory compartment is not yet known. However, it appears to involve the regulated shut-down and silencing of latent EBV gene expression from an initial state called latency III or the growth programme (as found in LCLs), via latency II (also known as the default programme), until in quiescent memory B cells no EBV proteins can be detected in a state called latency 0 (or the latency programme) (reviewed in [3]).

Using recombinant viruses established with a bacterial artificial chromosome (BAC) system, we recently showed that EBNA3A and EBNA3C are both necessary for repression of the cellular gene *Bim* [4]. The Bim protein (Bcl-2 interacting mediator of cell death) is a pro-apoptotic BH3-only, Bcl-2-family member that appears to be a uniquely important tumour suppressor in the development of B and T lymphocytes. It regulates apoptosis during lymphocyte development by binding and inactivating pro-survival members of the Bcl-2 family and binding and activating the pro-apoptotic family member Bax (reviewed in [5],[6]). The role of Bim in lymphomagenesis came sharply into focus when it was discovered that in Eμ-*Myc* transgenic mice constitutively expressing Myc in B cells, loss of even a single *Bim* allele significantly accelerated lymphoma development and revealed *Bim* as a haploinsufficient tumor suppressor [7]. Deregulation of *Myc* through reciprocal chromosome translocations that put the gene under the influence of immunoglobulin locus control elements is a hallmark of all BLs (reviewed in [8],[9]). The importance of Bim in a cell carrying a deregulated *Myc* became apparent when it was discovered that under these conditions combined activation of both the ARF/p53 pathway and *Bim* leads to apoptosis [10]. However, when Myc is mutated or either the activation of ARF/p53 or *Bim* is impaired, the result is B lymphomagenesis [10],[11]. The clear implication is that if EBV inhibits an increase in Bim expression when wild-type *Myc* is deregulated by translocation, this could be a mechanism through which EBV directly contributes to the development of BL.

Since during latency III, EBNA2 constitutively activates *Myc*, this repression of Bim expression is probably critical for the immortalisation of B cells by EBV, persistence *in vivo* and perhaps the development of the endemic EBV-positive form of BL [4],[12] – it is therefore central to EBV biology. However the details of how Bim levels are modulated by EBV is a controversial subject since it has been reported that EBV can alter both *Bim* gene expression and Bim protein stability [4],[13]. Here the molecular mechanism by which EBV regulates the amount of Bim has been explored further and this has revealed that heritable, epigenetic modifications in the 5′ regulatory region of *Bim* play a major role in determining the level of Bim protein expressed in EBV infected B cells.

## Materials and Methods

### Ethics statement

This study was conducted according to the principles expressed in the Declaration of Helsinki. The samples for this study were obtained from the archives of the VU University medical centre. These were collected during 1996–2007 as part of collaborative studies in Malawi and Uganda on the diagnosis of Epstein-Barr virus associated malignancies. Written, informed consent was obtained from the guardians of study participants at the time of collection.

### Cell culture

All B cell lines were cultured in RPMI-1640 medium (Invitrogen) supplemented with 10% fetal calf serum, penicillin, streptomycin, 1 mM sodium pyruvate (Sigma) and 50 µM α-thioglycerol (Sigma). 100 µg/ml hygromycin B (Roche) was added to cultures of BL containing recombinant hygromycin- resistant EBV (BL31 WT, BL31 E2KO), as described in [4]. All other cell lines used in this study were described previously in [14],[15],[16]. 24 hours before any experimental treatment, cells were seeded at a density of 2.5×10^5^ cells/ml.

### Cell treatments

MG-132 (Calbiochem) was used at a final concentration of 5 µM, 10 µM or 15 µM. Actinomycin D (Sigma) was used at a final concentration of 5 µg/ml. Trichostatin A (TSA), 5-azacytidine (AZA) and sodium butyrate were all purchased from Sigma and were used at concentrations of 500 nM, 5 µM and 2.5 mM respectively.

### Western immunoblotting

Western immunoblotting was performed as described previously [4],[15],[17]. Primary antibodies used for western blot probing were: rabbit polyclonal anti-Bim/BOD (Stressgen, AAP-330), mouse monoclonal anti-p21^WAF1^ (SX118, kind gift from Prof. Lu Xin, Ludwig Institute, Oxford), mouse monoclonal anti-γ-tubulin (Sigma, T6557), mouse monoclonal anti-BZLF1 (kind gift from Prof. Paul Farrell, Imperial College London), sheep polyclonal anti-EBNA3A (Exalpha, USA), mouse monoclonal anti-EBNA3C (A10, kind gift from Prof. Martin Rowe, University of Birmingham).

### Quantitative reverse transcriptase real time PCR (Q RT-PCR)

For Q RT-PCR, RNA was extracted from approximately 5×10^6^ cells for each cell line using the RNeasy mini kit from Qiagen and following the manufacturer's instructions. To quantify Bim_EL_ mRNA after HDAC and DNMT inhibitors treatment, 25 ng of RNA were used for each reaction with one-step QuantiTect SYBR green RT-PCR kit from Qiagen and ABI PRISM 7700 Sequence Detector, according to the manufacturer's instructions. Bim_EL_ primers were GCTGTCTCGATCCTCCAGTG and GTTAAACTCGTCTCCAATACG as described previously [4]. GAPDH was used to normalize Bim_EL_ levels. GAPDH primers were purchased from Qiagen (Hs_GAPDH_2_SG; QT01192646). The PCR conditions were 30 min at 50°C, 15 min at 95°C and then 30 sec at 95°C, 30 sec at 58°C and 30 sec at 72°C for 40 cycles. The standard curve method was used. Standards were a mix of all RNAs used and five 10-fold serial dilutions of this.

For quantification of mRNA after actinomycin D treatment, 5 ng of RNA were used for each reaction with one-step QuantiTect SYBR green RT-PCR kit from Qiagen and ABI PRISM 7900 Sequence Detector, according to the manufacturers' instructions. The same primers as above were used for Bim_EL_. For Myc the primers were AGCTGCTTAGACGCTGGATTT and GAGGTCATAGTTCCTGTTGGTGAA and for β-Actin they were GATGGAGTTGAAGGTAGTTTCGTG and GCGGGAAATCGTGCGTGACATT [18]. Reaction conditions were the same as above. The calculated errors in the graphs are the standard deviations from three replicate Q RT-PCR reactions for each mRNA.

### Chromatin immunoprecipitations (ChIP)

Chromatin Immunoprecipitation Assay Kit from Upstate (17-295) was used, according to the manufacturer's protocol. To obtain sheared chromatin with DNA of 200 bp–1000 bp in length, extracted chromatin from 1×10^6^ cells per ChIP was sonicated in 200 µl lysis buffer for four 20 sec sonication rounds, using a Heat Systems Sonicator Ultrasonic Processor XL at 10% intensity.

The antibodies for the acetylated histones IPs were rabbit polyclonal IgG anti-acetyl-Histone H3 (Upstate; 06-599), rabbit polyclonal IgG anti-acetyl-Histone H4 (Upstate; 06-866) and as a negative control Rabbit IgG serum (Upstate; PP64). Precipitated DNA was assayed by quantitative PCR (Q-PCR) using Qiagen's QuantiTect SYBR green PCR kit. 2% of input was compared to the IP sample and the values from the IgG negative control were subtracted as background. Primers for *Bim* were CTGGTCTGCAGTTTGTTGGA and GGTGGCTGCAAGAATCAAGT. *β-Actin* was used as an independent control. *β-Actin* primers were TGCACTGTGCGGCGAAGC and TCGAGCCATAAAAGGCAA [19]. The PCR conditions were 15 mim at 95°C and then 20 sec at 95°C, 30 sec at 55°C, 30 sec at 72°C for 40 cycles, using ABI PRISM 7700 Sequence Detector.

ChIP for histone H3 trimethylated at K27 was performed as described above, using anti-H3K27-Me3 antibody from Upstate (17-625) and as a negative control Rabbit IgG serum (Upstate; PP64). The primers used to quantify precipitated DNA were: pair A: TTTAGAAAGAATCTTGGCAGTCAACTCCTC and CAATGGCTGGTGAAAAGGAGGGTTT, pair B: GAAGGACCAGGGAGGAAGGACCAAG and TGACACCTAGCCCAGTGGAAACCCC, pair C: CGAGCGGGAAAAAAGGTTTGGTTCA and TAGGCTCCCACTTCCTTCTCCCAGT, pair D: AAGAGCAAAGTTCGTCCGCGGTAGG and TATTTCGCTGCAAGAGGGAAAAGGCAC, pair E: GACCCTCAGAGGGAGGAGAGCTCAAA and GCCCTGAGTTTCTAAGCCGCTCTGG, pair F: CGCCAGCAGGCAGAGTTAC and CAGGCTCGGACAGGTAAAGG. The cycling conditions were 2 min at 50°C, 10 min at 95°C and then 15 sec at 95°C, 1 min at 60°C for 40 cycles, using ABI 7900 384-well Real-Time PCR machine. The calculated errors in all graphs presenting ChIP data are the standard deviations from three replicate Q-PCR reactions for precipitated chromatin, input chromatin and background (chromatin precipitated with non-specific rabbit IgG).

### Methylation specific PCR (MSP) and bisulphite sequencing

For the methylated DNA control, *in vitro* methylated Jurkat DNA was used (Active Motif, 102163). B cells were isolated from a buffy coat residue purchased from the blood transfusion service, using the AutoMACS 3 sorting system from Millitenyi Biotec. CD19 positive selection, with the CD19 MicroBeads from the same manufacturer (130-050-301), was performed according to their instructions. PBMC genomic DNA was extracted from blood of a healthy donor. Genomic DNA was extracted with the Qiagen DNeasy Kit for all the cells described. 200–500 ng of genomic DNA was converted by bisulphite using EZ DNA methylation kit by Zymo Research (D5002). For MSP the converted DNA was amplified using 5 sets of primers, specific for 5 different regions of the *Bim* promoter. The sequences were – For Set I: U2F: GTTTTGGGGTTTTGTAAGGAAAT; U2R: AAATAATAAAAATATTTTCCACACC; M2F: TTTTGGGGTTTTGTAAGGAAAC; M2R: TCAAATAATAAAAATATTTTCCGCG. For Set II: U3F: GGTTTAGTTTTGGGAGGATTTTTT; U3R: ACCAAAAATAACAAATTCATACATC; M3F: GTTTAGTTTCGGGAGGATTTTTC; M3R: ACCAAAAATAACGAATTCATACGTC. For Set III: U4F: TTTTTGAGTTTATTTAGTTGTGTTTATGT; U4R: AAAACCTTAAATTCCCAAAATCACT; M4F: CGAGTTTATTTAGTCGTGTTTACGT; M4R: CTTAAATTCCCGAAATCGCT. For Set IV: U1F: TGTTTAAAATTTATTTGAAAATGTGT; U1R: ACAAATAAAAAAACATTATCCCACC; M1F: TCGTTTAAAATTTATTCGAAAATGC; M1R: AACAAATAAAAAAACGTTATCCCG. For Set V: U5F: GTAGATTTAGAGGATTGGAGAGGTG; U5R: ACCAATAAAAACAAAACAACTAAATTCA; M5F: GTAGATTTAGAGGATTGGAGAGGC; M5R: CAATAAAAACAAAACAACTAAATTCGA.

For bisulphite sequencing primers AAAACCCCCAAAATTTACTAAACTC and GGGAATTTAAGGTTTTTTTTATTT were used to amplify a region of the *Bim* promoter (see below), in order to sequence and study the methylation state of 36 CpG dinucleotides in this region. The amplified PCR products were cloned using the Invitrogen TA cloning kit. Plasmids containing the cloned sequences were amplified by rolling circle amplification (Templiphi; GE healthcare) and sequenced. Between 8 and 24 clones were sequenced for each cell line.

### BL biopsy samples

Frozen tissue was collected in parallel with routine biopsy specimens from suspected BL-cases at the Uganda Cancer Institute by Dr. J.Orem (Makarere University, Kampala, Uganda) and were retrieved from the archives of Dept. Pathology, VU University medical centre in Amsterdam, The Netherlands. All biopsies were classified by routine cytology on paraffin-embedded formalin-fixed tissue and EBER-RISH using PNA probes (DAKO, Glostrup, Denmark) [20].

### Precipitation of methylated DNA with His-tagged MBD2b

After isolating genomic DNA as described above, 2 µg of DNA was sonicated in 200 µl of H_2_O for five 20 sec sonication rounds, using a Heat Systems Sonicator Ultrasonic Processor XL at 10% intensity, to produce sheared DNA with length of 200–1000 bp. Methylated DNA was precipitated from 1 µg of sheared DNA using the MethylCollector kit (Active Motif, 55002) according to the manufacturer's instructions. Precipitated DNA was quantified in exactly the same way as described for the ChIP for H3K27-Me3, with the same primer pairs (A–F), the same quantitative PCR conditions and the same method of analysis. As background control, precipitations with just the magnetic beads were performed, without addition of His-MBD2b. As additional controls, precipitations were performed using DNA from PBMCs (unmethylated) and Jurkat DNA (fully methylated *in vitro*). The calculated errors in all graphs presenting methylated DNA precipitation data are the standard deviations from three replicate Q-PCR reactions for precipitated DNA and input DNA.

## Results

### Latent EBV does not suppress Bim protein levels by enhancing proteasome-mediated proteolysis or reducing Bim mRNA stability

**Figure 1:**
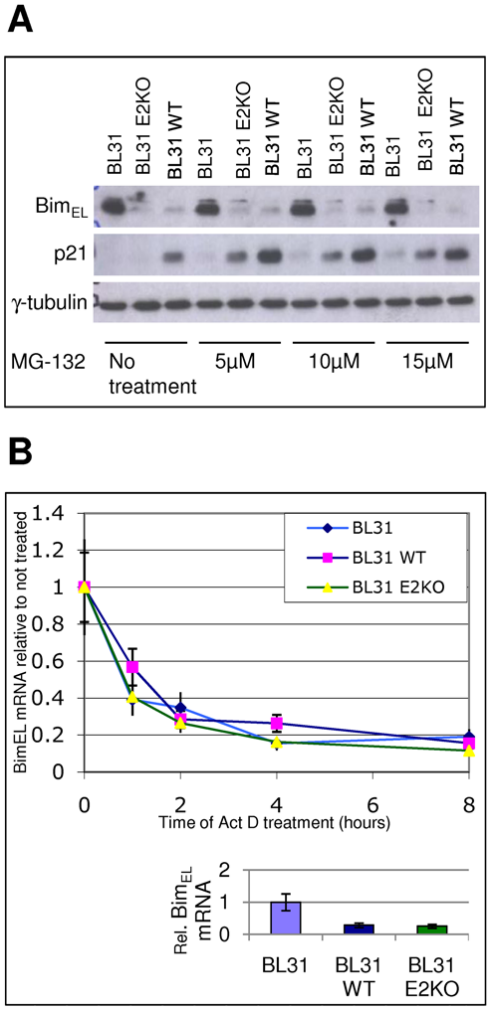
Down-regulation of Bim in EBV-positive cells is not due to proteosomal degradation or decreased mRNA stability. A) Uninfected EBV-negative BL31 cells, or BL31 cells infected with a wild type (B95.8 strain) EBV-BAC (BL31 WT), or BL31 infected with an EBV-BAC carrying a deletion of the EBNA2 gene (BL31 E2KO) were treated with the proteasome inhibitor MG-132 for 8 hours at the concentrations indicated. Protein levels were assessed by western immunoblotting. p21^WAF1^ protein was used as a control and its accumulation after treatment in all cells indicates that proteasome-mediated degradation is inhibited. Bim_EL_ levels are unaffected and remain low in EBV infected cells. B) The same panel of cells was treated with the inhibitor of transcription actinomycin D (5 µg/ml) for 8 hours. Bim_EL_ mRNA levels were measured by quantitative RT-PCR, using constant amounts of total RNA for each time point, and are shown in the graph relative to the starting Bim mRNA levels. The amounts of Bim_EL_ mRNA at the beginning of the treatment, relative to the levels in uninfected cells, are shown on the column graph at the bottom. The rate of mRNA degradation is similar in all three cell-lines.

It has been reported that EBV reduces Bim expression by targeting it to the proteasome system for degradation [13]. Since EBV induces the phosphorylation of extracellular signal regulated kinase (ERK) and this activated form of ERK can phosphorylate Bim and mark it for ubiquitinylation, it was suggested that latent EBV enhances Bim turnover by proteasomes. However, we recently showed that inhibition of ERK phosphorylation in EBV-positive BL cells does not necessarily result in significantly higher levels of Bim, but that in EBV-positive cells Bim mRNA was always substantially reduced [4]. Nevertheless, this does not exclude the possibility that Bim protein is degraded by the proteasome system via another signalling pathway. In order to investigate this in more detail, EBV-negative BL31 cells, BL31 cells infected with a wild type (B95.8 strain) EBV-BAC (BL31 WT) and BL31 infected with an EBV-BAC with EBNA2 deleted (BL31 E2KO) were treated with the inhibitor of proteasome function, MG-132. Western blots in Figure 1A show that the addition of increasing amounts of MG-132 had no significant effect on the expression of the major Bim isoform Bim_EL_ after eight hours. The same was true after twenty-four hours of treatment, however at this time there was considerable cell death (data not shown). Under the same conditions p21^WAF1^ – a protein with a relatively short half-life because it is rapidly degraded by proteasomes [21],[22] – accumulated, thus demonstrating that the MG-132 successfully inhibited proteosome-mediated proteolysis. It is unlikely therefore that enhanced protein degradation is the main cause of the lower levels of Bim seen in B cells containing latent EBV. Although latent EBV gene expression can alter ERK signalling and may therefore affect Bim by phosphorylation and ubiquitinylation, our data are consistent with EBV primarily inhibiting the *de novo* synthesis of Bim, rather than enhancing its turnover.

Recently it was shown that Bim levels are also regulated by modulation of Bim mRNA stability [23]. It is therefore conceivable that EBV reduces the stability of Bim mRNA, leading to lower steady-state levels of Bim transcripts and protein in infected cells. To examine this possibility, the same BL31 cell lines were treated with the inhibitor of transcription actinomycin D for up to eight hours to block *de novo* synthesis of RNA. Bim_EL_ mRNA was then analysed by quantitative (Q) RT-PCR to assess whether in EBV infected cells Bim_EL_ mRNA was degraded more rapidly (Figure 1B). Although, as expected, the starting levels of Bim mRNA were different in each cell line (see histogram in Figure 1B), in each cell line the mRNA levels dropped, showing that transcription was successfully inhibited. Moreover, the rate of Bim_EL_ mRNA degradation was very similar in EBV infected and uninfected cells indicating that the presence of latent EBV does not significantly influence Bim_EL_ mRNA stability. In parallel, the stability of an mRNA known to have a short half-life (Myc) and one known to be very stable (β-Actin) were assessed (Figure S1).

The data from these experiments using inhibitors of either proteosome enzymes or mRNA synthesis are therefore most consistent with the regulation of Bim expression occurring principally at the level of transcription as we previously suggested [4].

### Inhibitors of HDACs and DNMTs increase Bim_EL_ protein and mRNA levels in EBV infected cells

**Figure 2:**
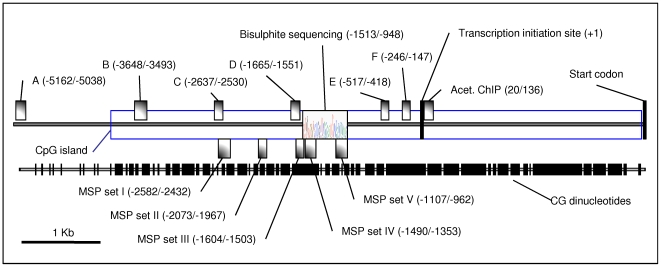
Schematic of the *Bim* promoter and CpG-island. The *Bim* 5′ regulatory region has a very large CpG-island that could be subject to transcriptional control. The predicted CpG-island is 6718 bp long and contains 595 CpG dinucleotides. The regions amplified by PCR for chromatin immunoprecipitation analysis [ChIP, (A–F)] and methylation specific PCR [MSP, (I–V)] and the region sequenced after bisulphite conversion (bisulphite sequencing) are shown, with their positions relative to the transcription initiation site indicated. The locations of the predicted transcription initiation site and start codon are indicated [54].

In the absence of evidence supporting an alternative mechanism for Bim regulation by EBV, we investigated whether latent EBV could influence transcription of *Bim* via the modification of local chromatin. There are several reports of Bim expression being regulated by modulation of transcription (for examples see [24],[25],[26]) and a striking feature of the gene is an unusually large CpG island of more than 6000 bp extending either side of the transcription initiation site (http://genome.ucsc.edu/cgi-bin/hgGateway; Figure 2); this region could be subject to control by DNA methylation. Since both histone deacetylation and DNA methylation are associated with repressed, inactive chromatin, these were investigated initially using chemical inhibitors (reviewed in [27],[28],[29]).

**Figure 3:**
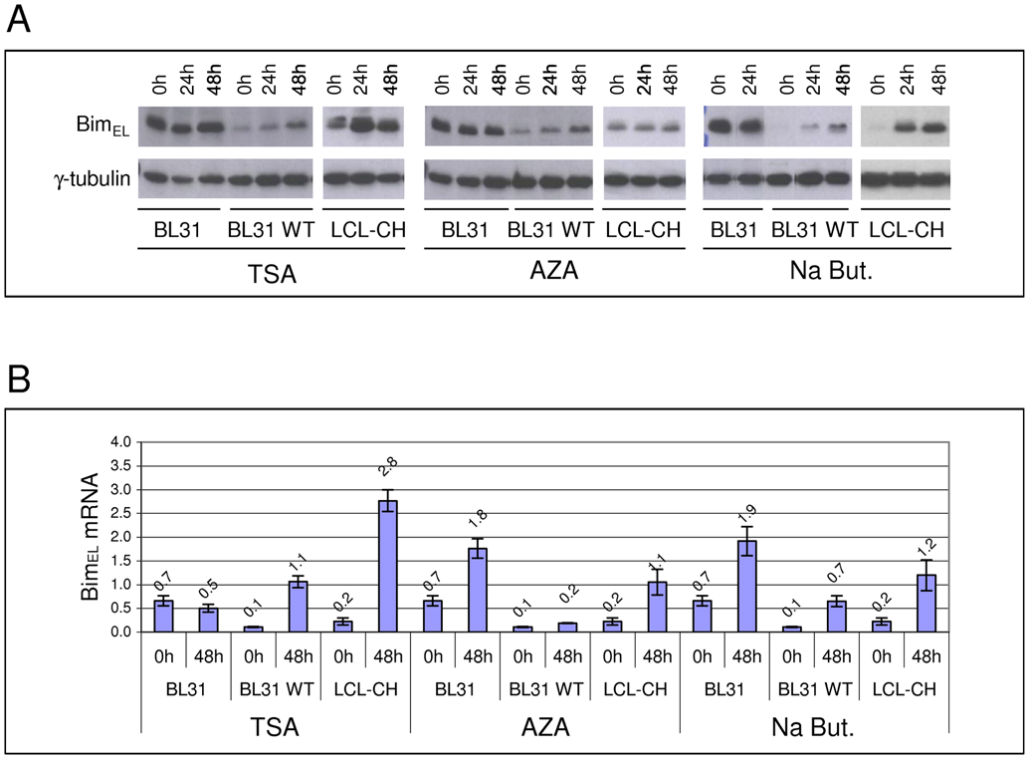
Bim expression can be up-regulated in EBV infected cells after treatment with HDAC or DNMT inhibitors. A) EBV-negative BL31, EBV-positive BL31 WT and LCL-CH were treated with TSA (500 nM), 5′ azacytidine (AZA; 4 mM) or sodium butyrate (Na But; 2.5 mM) for up to 48 hours. Bim_EL_ protein levels were assessed by western immunoblotting. B) After similar treatments Bim_EL_ mRNA levels were measured by quantitative RT-PCR. Bim_EL_ mRNA levels were all normalized with GAPDH mRNA levels. The values over the columns indicate their height, which represents abundance of Bim mRNA relative to the standards used, after normalization with GAPDH mRNA levels.

To get an indication of whether the regulation of *Bim* transcription might involve local chromatin or DNA modifications BL31, BL31 WT, and LCL-CH cells were treated with the HDAC inhibitor trichostatin A (TSA), the DNMT inhibitor 5′ azacytidine (AZA) or with sodium butyrate – which is reported to suppress HDACs and might affect DNA methylation [30],[31]. Cells were treated with one of the inhibitors and samples were taken after 24 and 48 hours. The levels of Bim_EL_ protein following these treatments can be seen in western blots (Figure 3A). Exposure of EBV-positive BL31 WT cells to TSA resulted in a significant up-regulation of Bim_EL_ levels, but in uninfected BL31 cells the already high level remained unchanged. In the LCL cells up-regulation was more pronounced – even after 24 hours of treatment. AZA had more moderate effects on Bim_EL_ protein levels, with little change in the LCL, but significant up-regulation in BL31 WT. Exposure of both BL31 WT and the LCL to sodium butyrate produced the most substantial increase of Bim_EL_. Two other EBV negative/positive “pairs” of BL cell lines, BL41/BL41 B95.8 and Ramos/Ramos-AW together with two BL-derived cell lines Eli and Akata 6, were treated in a similar way and these showed the same trend of Bim_EL_ up-regulation as described above (Figure S2A and B). Since both TSA and AZA have been shown to induce the lytic cycle in EBV infected cells [32],[33], western blots were performed to determine whether the EBV lytic switch protein BZLF1 was induced. No correlation between lytic cycle and Bim expression was detected in these cells (Figure S2C). This is consistent with our previous observations [16].

Subsequently the amount of Bim_EL_ mRNA in cells harvested after similar treatments was assessed by real-time Q RT-PCR (Figure 3B). This analysis was performed on samples taken 48 hours after the addition of the inhibitor. Bim_EL_ sequences were amplified using primers described previously [4]. Values for Bim_EL_ mRNA were normalized to values obtained for GAPDH primers. Quantification used standard curves for each pair of primers, always using the same standards. Similar trends of Bim_EL_ up-regulation were seen at the mRNA level (Figure 3B) as at the protein level (Figure 3A), indicating that latent EBV is associated with repression of *Bim* transcription via mechanisms involving both histone deacetylation and DNA methylation. An exception was LCL-CH in which the up-regulation of Bim mRNA by 5-azacytidine was significantly greater than the amount of protein induced. We do not know the reason for this discrepancy, but it should be noted that all three inhibitors were very toxic in the cells used here, inducing a great deal of cell death and so making these experiments rather unsatisfactory. Nevertheless the results were each largely consistent with the hypothesis that EBV repression of *Bim* involves local chromatin modifications.

### EBV reduces acetylation of histones occupying the *Bim* promoter

**Figure 4:**
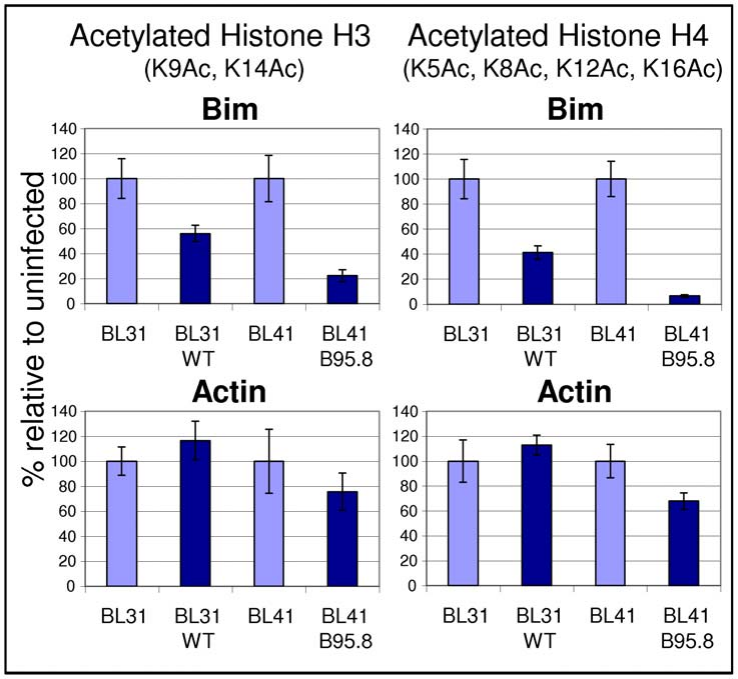
Latent EBV decreases occupancy of acetylated histone-H3 and histone-H4 on the *Bim* promoter. ChIPs were performed on EBV-negative BLs (BL31 and BL41) and BLs infected with a wild type EBV-BAC (BL31 WT) or virus (BL41 B95.8), using antibodies against acetylated histone H3 (K9Ac, K14Ac) and acetylated histone H4 (K4Ac, K7Ac, K11Ac, K15Ac). Immunoprecipitated DNA, associated with acetylated histones, was assayed by quantitative PCR and expressed in the graphs as fold enrichment relative to input chromatin. Changes are specific to *Bim*, since occupancy of acetylated histones on the *Bim* promoter decreases significantly in EBV-infected cells (upper panels). Occupancy of β-*Actin* promoter does not change significantly whether or not the cells carry EBV (lower panels).

Since Bim levels can be increased in EBV infected cells by treatment with HDAC inhibitors, it seems likely that EBV regulates *Bim*, at least in part, through the modulation of histone acetylation. If the effect is direct, this means that in the presence of EBV, histones associated with the *Bim* promoter should be less acetylated relative to uninfected cells. To directly assess occupancy of the *Bim* promoter by acetylated histones, chromatin immunoprecipitations (ChIPs) were performed using antibodies directed against acetylated histone H3 (K9Ac, K14Ac) and acetylated histone H4 (K4Ac, K7Ac, K11Ac, K15Ac). The results are shown in Figure 4. Real-time Q PCR was used to measure the abundance of a DNA fragment from the *Bim* promoter that was associated with immunoprecipitated histones. The primers used correspond to a region just downstream of the transcriptional start site (shown in Figure 2). Acetylated histone occupancy of the β-*Actin* promoter was measured as a control. Values for each immunoprecipitation were normalized to 2% of input DNA, and in each case the values obtained from the control sample incubated with rabbit pre-immune IgG were subtracted as background. Two EBV-negative BL lines were used, BL31 and BL41, and their EBV-positive counterparts, BL31 WT and BL41 B95.8. It is clear that EBV significantly decreases acetylated histone (H3 and H4) occupancy in both EBV positive lines, relative to the uninfected ones. Histone acetylation on the β-*Actin* promoter was largely unaffected by EBV, indicating that the effect on *Bim* is specific. The data are consistent with EBV inhibiting *Bim* transcription by the direct modulation of biochemical marks on chromatin of the *Bim* promoter.

### Latent EBV increases DNA methylation of the *Bim* promoter

DNA methylation is associated with inactive chromatin and the repression of transcription [27],[28],[29],[34]. The observation that treatment of some EBV-infected B cells with the DNMT inhibitor 5′ azacytidine led to the de-repression of Bim, and the presence of a remarkably large CpG island spanning the 5′ regulatory region of *Bim*, led us to investigate the DNA methylation status of the *Bim* promoter region. In order to discover whether EBV directly influenced DNA methylation patterns on the promoter we used methylation specific PCR (MSP) and bisulphite sequencing directed at various sites upstream of the transcription initiation site (see schematic in Figure 2). For the initial analysis, twenty-six cell lines (plus primary cells) were investigated by MSP and of these, twenty were studied further using bisulphite sequencing to determine the DNA methylation state of *Bim* relative to their EBV status (ie EBV-positive or -negative). The EBV-negative cells used were either primary cells [peripheral blood mononuclear cells (PBMCs) or purified CD19^+^ primary B cells] or EBV-negative BL cell lines. The EBV-positive cell lines were LCLs (early passage or late passage), *in vitro* BL ‘converts’ (that is BL lines established from EBV-negative tumours and then infected *in vitro* with EBV), or BL lines established from EBV-positive tumours.

**Figure 5:**
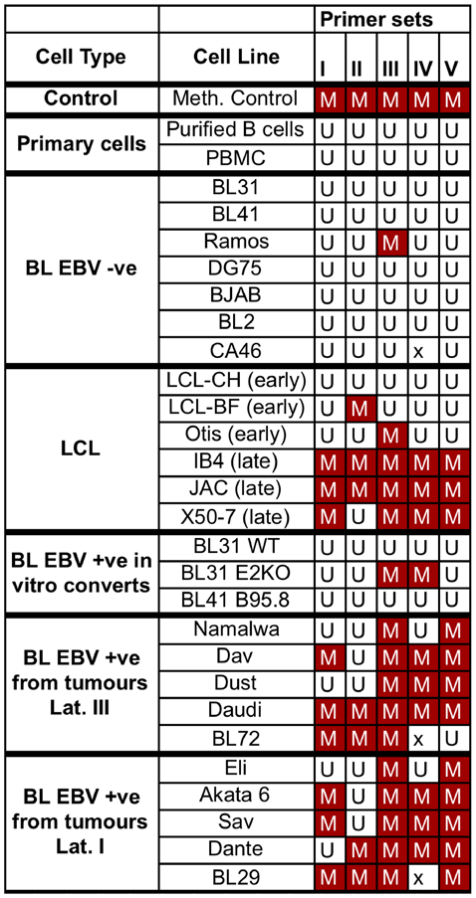
MSP analysis of the *Bim* promoter in EBV-negative and EBV-positive cells. Five different sets of methylation specific primers were used for five different regions of the *Bim* promoter. In the matrix presented, the DNA methylation state assessed with the MSP primers is indicated for each primer set used. When an amplification product was apparent with the primers specific for unmethylated DNA, but not with the primers specific for methylated DNA of the same region, the cell line was scored as unmethylated (U in colourless boxes) at the regions the MSP primers anneal. When an amplification product was apparent with the primers specific for methylated DNA the cell line was scored as methylated (M in red boxes), for the relevant regions. X indicates that MSP was not performed with this primer set on this sample. DNA isolated from peripheral blood mononuclear cells (PBMC) and purified primary B cells was used as control for unmethylated DNA. *In vitro* methylated DNA from Jurkat cells was used as control for methylated DNA (Meth).

For the MSP analysis five sites were tested with primer sets (I–V) of two primer pairs specific for either methylated or unmethylated DNA. The regions of the *Bim* promoter amplified by the MSP primers are indicated in Figure 2. The results are summarized in the matrix shown in Figure 5. Cell lines that gave a band with any of the methylation-specific primers were scored as methylated (M). Cell lines that only gave a band with primers for unmethylated DNA, and none with primers for methylated DNA were scored as unmethylated (U). These analyses were generally performed at least three times and an example of representative primary data for twenty cell lines plus primary cells using primer set III can be seen in Figure S3.

In PBMCs and purified primary B cells *Bim* was always completely unmethylated in these assays – as would usually be expected of a CpG-island associated with an active gene and protected by transcription factors from DNA methylation [34]. Similarly, all the established EBV-negative BL lines (that had all been cultured on and off for decades) were found to be unmethylated at these sites, with the exception of Ramos, which gave a faint band for methylation only with primer set III. In contrast, all late passage LCLs that have also been cultured for many years, showed methylation at nearly all these sites. One of the early passage LCLs (LCL-CH, recently established in our lab by the infection of CD19+ B cells with B95.8 strain of EBV [15] and only grown continuously in culture for 2–3 months before being frozen) was found to be unmethylated. EBV-positive *in vitro* BL converts gave a mixed picture, with two being methylated and one unmethylated. In contrast, all the BL lines established from EBV-positive tumours were methylated at two or more sites on the *Bim* promoter. This group included four BL cell lines exhibiting a latency I pattern of EBV gene expression. These latency I cells do not express EBNA3A or EBNA3C, the viral proteins that appear to be necessary for the initial down-regulation of Bim. This strongly suggests that DNA methylation of the *Bim* promoter is a secondary epigenetic effect of latent EBV infection and may be triggered in BL progenitor cells (for a more detailed and considered discussion of this point see below).

**Figure 6:**
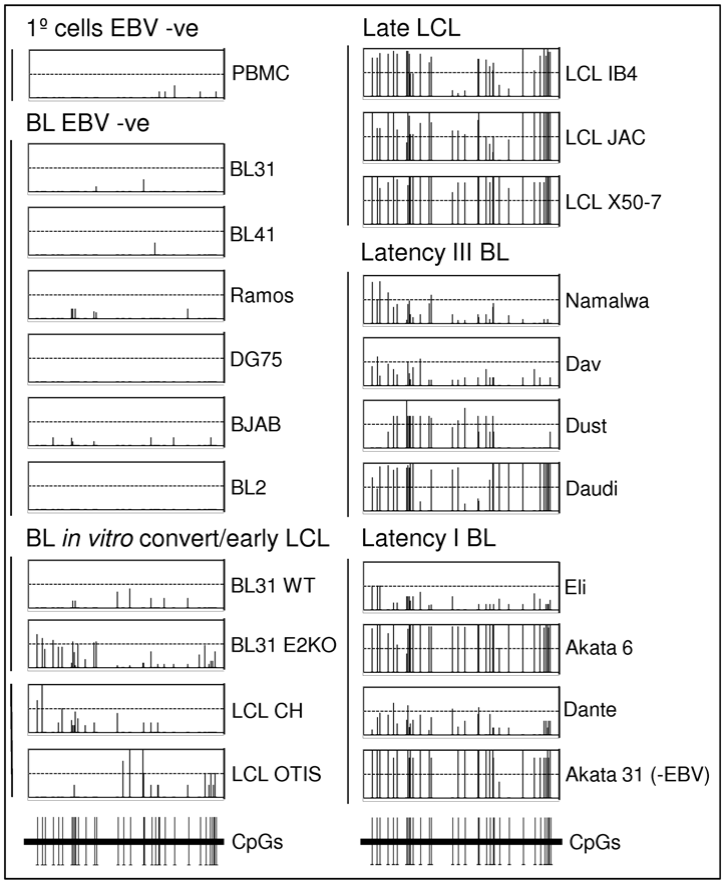
Bisulphite sequence analysis of the *Bim* promoter from cell lines. A region of 565 bp was sequenced and 36 CpGs were scored within this for their methylation state. An average of 12 clones for each cell line were analysed. The position of the bars on the X-axis within each box exactly corresponds to the position of the CpG in the sequenced region. The height of the bars reflects the percentage of sequenced clones found to have a methylated cytosine at that particular CpG dinucleotide. If the bar reaches the top of the box all clones were methylated at the particular CpG (100%). All EBV-positive cell lines appear to be significantly more methylated than all the EBV-negative cells. EBV-positive cell lines shown include *in vitro* converts, early and late passage LCLs and tumour-derived cell lines with either latency III or latency I patterns of EBV expression. In the bottom right panel results for Akata 31 are shown, a cell line that was derived from a culture of latency I Akata, but that has lost the EBV episome.

Bisulphite sequencing was used for a more comprehensive investigation of the CpG methylation pattern on the *Bim* promoter of twenty-one cell lines plus the PBMCs. After bisulphite modification, a region of 565 bp was subjected to DNA sequence analysis and the 36 CpG dinucleotides were scored for their methylation state. An average of 12 and a minimum of 8 clones were sequenced for each cell type. The results presented in Figure 6 give a detailed “snapshot” of the DNA methylation state of a significant region of the *Bim* promoter and CpG-island. The data are largely consistent with and extend the MSP data that are summarized in Figure 5. PBMCs and all the EBV-negative BL lines are essentially unmethylated across this region of the *Bim* promoter. In contrast to these EBV-negative cells, all the EBV-positive cell lines show a significantly higher frequency of methylated CpGs. Nevertheless, again the BLs infected with EBV *in vitro* and early passage LCLs are generally less methylated than late passage LCLs and all the EBV-positive BL lines derived from EBV-positive tumours. These and the MSP data are consistent with epigenetic repression via chromatin modification preceding DNA methylation as has been described previously for other genes [35],[36]. Included in the series that were subjected to bisulphite sequencing was a sub-clone of Akata BL (Akata 31 [37]) that has lost the EBV episome in culture and therefore expresses no EBV factors. The *Bim* promoter in Akata 31 appears to be almost as methylated on CpG dinucleotides as the EBV-positive clone Akata 6, indicating that – once it is established – DNA methylation of this locus does not require EBV for its maintenance.

### Methylation of the *Bim* promoter in BL biopsy samples

**Figure 7:**
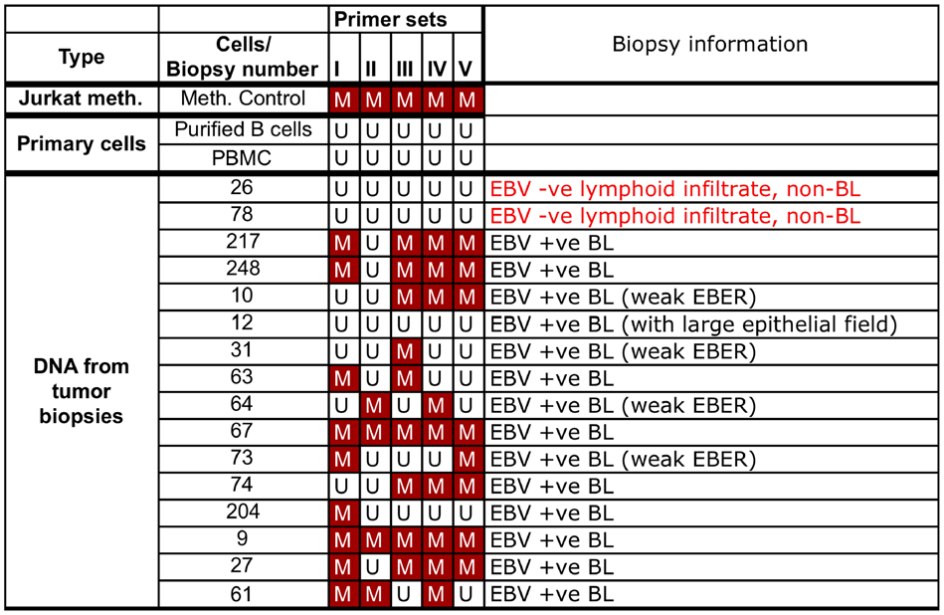
*Bim* promoter DNA is methylated in biopsies from EBV-positive BL. MSP was performed for DNA isolated from biopsy material, in the same manner as with DNA from cell cultures (Figure 6 and Materials and Methods). In the matrix presented, the methylation state of the *Bim* promoter is shown for the regions assessed with each of the 5 MSP primer sets used. CpG methylation is indicated with “M” in red boxes. The unmethylated state is indicated with “U” in colourless boxes. The EBV status for each biopsy was determined by microscopy and EBER RISH and is indicated on the right panel (see also Figure 8 for representative examples). *In vitro* methylated DNA (Meth) and primary cells (PBMCs and B cells) were used as positive and negative controls respectively. Two EBV-negative, lymphoid infiltrates showed no evidence of DNA methylation by MSP at the *Bim* promoter.

**Figure 8:**
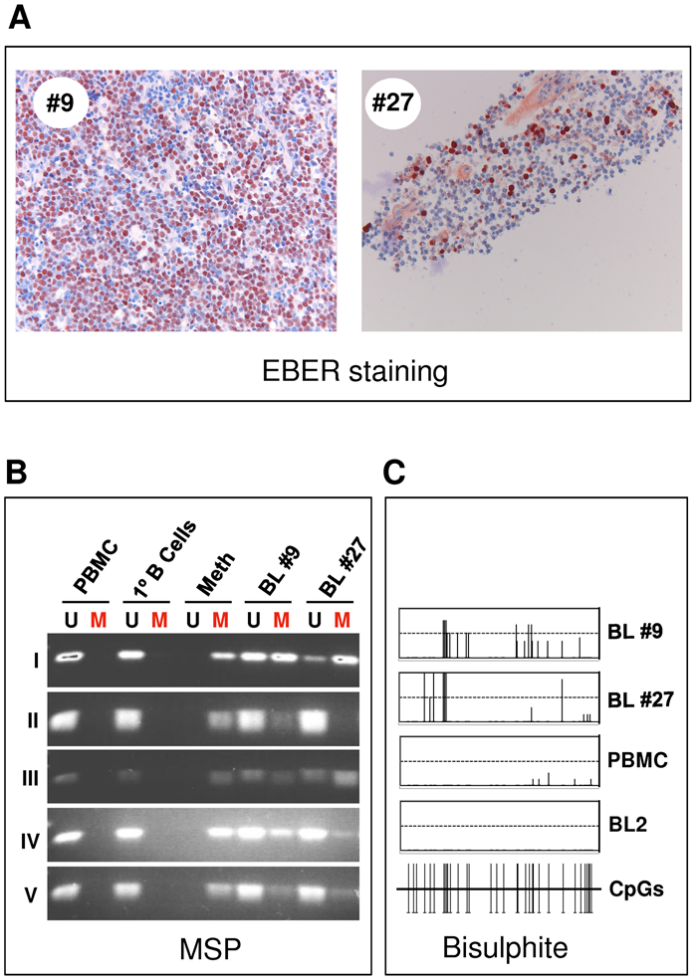
DNA methylation analysis of the *Bim* promoter by MSP and bisulphite sequencing in two EBER-positive BL biopsies. A) EBER RISH was performed to confirm the presence of EBV in tumour material. Cells staining brown-pink are positive for EBER RNA. B) MSP was performed for DNA isolated from the biopsies, in the same manner as with DNA from cell cultures (Figure 6 and Materials and Methods). Amplified DNA was visualized by agarose gel electrophoresis, for all primer sets (I–V), with primer sets specific for unmethylated state (U) or methylated state (M). C) Bisulphite sequencing was performed on DNA from these two EBV-positive biopsies to study DNA methylation at higher resolution. The results of sequencing DNA from PBMC and BL2 are shown in the bottom two panels for comparison.

The same MSP primer sets used for all the B cell lines shown above were used to test the methylation state of the *Bim* promoter in DNA extracted from 14 randomly selected African BL biopsy samples (summarized in Figure 7). All these samples were isolated from patients with suspected BL and confirmed as true BL by routine pathological examination (monomorphic lymphoma with starry-sky macrophages) and a positive reaction by EBER-RISH, reflecting EBV presence in the tumour cells (see Figure 8A for two representative examples). DNAs isolated from purified CD19^+^ primary B cells and PBMCs were used as negative controls. As might be expected from biopsy-derived material, the results are rather heterogenous. Nevertheless the vast majority of BL (13/14) showed some evidence of *Bim* promoter DNA methylation by MSP. The only biopsy scoring completely negative was one that histopathology identified as including a large epithelial field, so the amount of BL-derived DNA from this sample is uncertain. Again the primary B cells, PBMC and in addition two EBV-negative lymphoid samples were totally negative for DNA methylation. DNA from two samples (9 and 27) was used for bisulphite sequencing and the results – together with EBER staining and MSP analysis – are shown in Figure 8. The level of methylation across the 565 bp stretch of sequenced DNA appears to be non-random, and at several loci 70–100% of the clones sequenced were positive for CpG methylation.

From all the biopsy samples, amplification products were also apparent with primers specific for unmethylated DNA (eg Figure 8B and Figure S4). This may be because in some of these rapidly proliferating tumours, at the time of harvesting, cells had just replicated their DNA and methylation was not established on the newly formed DNA strand. Alternatively, the unmethylated DNA could be the result of non-BL cells in the samples resulting from infiltration into the tumour or contamination during isolation of the biopsy samples. Nevertheless, the presence of amplification products with primers specific for the methylated state in most EBV-positive BL but not EBV-negative cells is consistent with the hypothesis that CpG methylation of *Bim* can occur during the pathogenesis of EBV-positive BL and is directed by EBV in some unidentified way.

### EBV increases trimethylation of histone H3 lysine 27 (H3K27-Me3) at the *Bim* promoter

The results of the DNA methylation studies on EBV-positive and -negative B cells suggested that CpG methylation was probably not the primary event in the suppression of *Bim* transcription by EBV. Specifically, early passage LCLs and EBV-negative BL cells newly converted by EBV infection *in vitro* have a reduced level of Bim (protein and transcripts) but show only very modest amounts of DNA methylation. In contrast late passage LCLs and BL cell lines derived from EBV-positive tumours have relatively high levels of DNA methylation that is non-randomly distributed on the *Bim* promoter.

**Figure 9:**
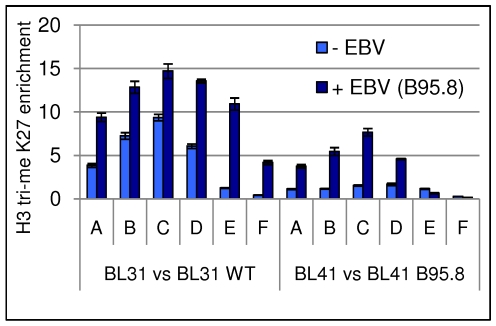
Increased levels of H3K27-Me3 along the *Bim* promoter in EBV infected cells. ChIPs were performed to study the occupancy of histone H3, trimethylated at K27, on the *Bim* promoter, relative to EBV status. The occupancy for BL31 and BL41 cells, infected with EBV or left uninfected are shown. Six primer pairs (A–F) were used corresponding to sites across the promoter/CpG-island region (see Figure 2 for locations). For each primer pair immunoprecipitated DNA associated with H3K27-Me3 was assessed by quantitative PCR and is expressed in the graph as fold enrichment relative to input chromatin. Trimethylated histone H3 at K27 is more abundant on the *Bim* promoter of EBV infected cells.

Since in cell transformation and oncogenesis, methylation of DNA is often preceded by trimethylation of lysine 27 on histone H3 (H3K27-Me3, [38],[39],[40],[41], reviewed [42]), we asked whether there were differences in this epigenetic chromatin mark on the *Bim* promoter in EBV-positive compared to EBV-negative B cells. Using the same two pairs of cell lines used for the histone acetylation ChIPs shown in Figure 4 and an essentially similar ChIP assay, but using mAbs directed against H3K27-Me3, the distribution of this mark across the *Bim* promoter was determined. The results shown in Figure 9 are consistent with the hypothesis that EBV targets *Bim* for repression via the methylation of H3K27. Using six sets of primer pairs (indicated as A–F in Figure 2) for amplification and real-time Q PCR analysis of various loci in the *Bim* promoter/CpG-island, it is clear that latent infection of both BL31 and BL41 with EBV is accompanied by a significant increase in H3K27-Me3 throughout most of the region. For reasons that are unknown the only primers sets that did not produce this differential effect were E and F when used on BL41 cells.

### Comparison of H3K27-Me3 and DNA methylation profiles throughout the *Bim* promoter

**Figure 10:**
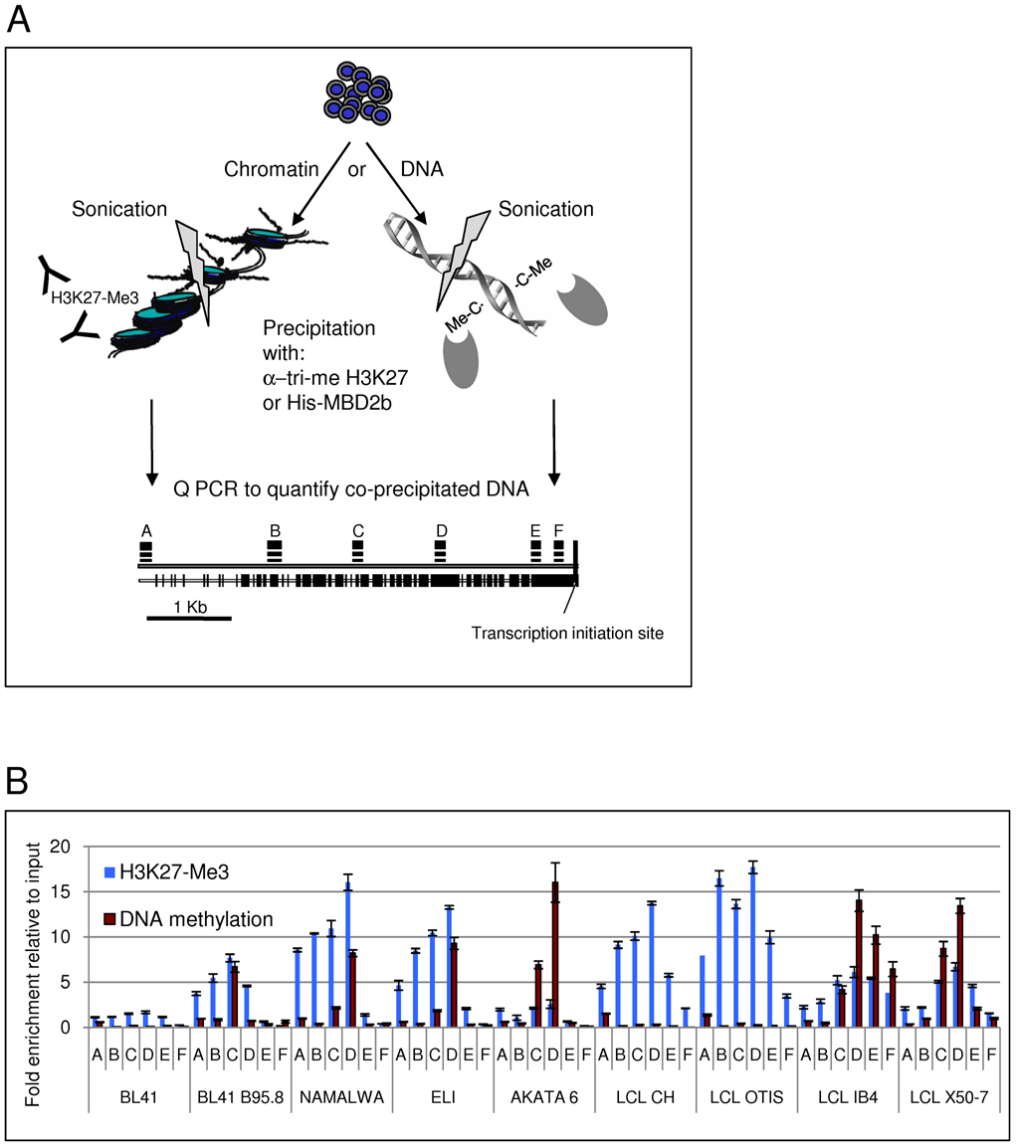
H3K27-Me3 and DNA methylation profiles along the *Bim* promoter. A) Schematic showing the analysis strategy. Chromatin and isolated DNA were sonicated and used for precipitation of either DNA associated with H3K27-Me3 or methylated DNA. The same 6 pairs of primers specific for loci spanning the *Bim* promoter (A–F, see Figures 2 and 9) were used to assess both H3K27-Me3 and DNA methylation in different cell lines. The correlation between these two epigenetic marks could thus be followed across the promoter region as indicated. B) BL41, infected with EBV or uninfected, latency III Namalwa, latency I Eli and Akata 6, early LCLs CH and Otis, and late LCLs IB4 and X50-7 were studied. The bars represent fold enrichment for co-precipitated DNA relative to input chromatin/DNA into each precipitation, as determined by quantitative PCR. The height of the bars is representative of the abundance of each epigenetic mark at the locus assessed by each primer set. The absolute abundance cannot be compared between the different epigenetic marks, because different precipitation methods were used (see Materials and Methods section). However, the trends for each mark across the promoter and between cell lines could be assessed.

In order to obtain greater insight into the relationship between H3K27-Me3 and CpG methylation at specific sites across the *Bim* promoter, we utilized a quantitative assay that depends on the precipitation of methylated DNA fragments by a histidine-tagged methyl-CpG-binding domain (MBD) protein (MBD2b). Using this technology it was possible to quantify the overall level of CpG methylation on the DNA fragments amplified by primers A–F (Figure 2 and 9) and in parallel measure the overall level of H3K27-Me3 at the same loci (see schematic in Figure 10A). These analyses were performed on BL41 and its EBV-positive equivalent, three cell lines derived from EBV-positive BLs (Namalwa, Eli and Akata 6) and two early passage LCLs (CH and Otis) that were compared with two late passage LCLs (IB4 and X50-7). The results are shown in Figure 10B. There was a trend in all the cell lines for the distribution of these epigenetic marks to be non-random. In general, the maximum amounts of both H3K27-Me3 and CpG methylation were associated with the loci amplified by primer sets C and D. Consistent with all the preceding data, cells expressing the EBV latency III pattern exhibited a significantly higher level of H3K27-Me3 than the EBV-negative and type I latency cells. The only anomaly was Eli, that was confirmed by western blotting to have retained its latency I phenotype (Figure S2D), but shows relatively high levels of H3K27-Me3 on the *Bim* promoter. One of the most striking observations from this series of experiments was that – as we saw using two other assays for CpG methylation (MSP and bisulphite sequencing) – in early passage LCLs (CH and Otis) the amount of DNA methylation on the *Bim* promoter was low. In contrast analysis of both late passage LCLs (IB4 and X50-7) showed *Bim* has become heavily methylated. Since Bim expression is similarly low in both early and late LCL cells, the repression of *Bim* transcription in LCL-CH and LCL-Otis is almost certainly associated with the very high levels of H3K27-Me3 distributed across the promoter region. The data are again consistent with the hypothesis that H3K27-Me3 on *Bim* precedes, and may then be replaced by the more stable epigenetic mark, DNA methylation.

## Discussion

EBV represses the expression of Bim in human B cells [4],[13],[16] and this study has revealed a remarkable correlation between latent infection of B cells with this gammaherpesvirus and repressive epigenetic marks on the chromatin of the *Bim* gene promoter. Bim is a highly regulated inducer of apoptosis, the level of which is a critical rate-limiting factor in B cell survival [5]. It is often deleted in mantle cell lymphoma [43] and in an Eμ-myc transgenic mouse model it is a haploinsufficient tumour suppressor [7] indicating that very modest changes in the amount of Bim in a B cell can have profound effects on its survival and the development of neoplasia. Bim levels are therefore finely regulated by modulation of transcription and modulation of both mRNA and protein stability (see Introduction). Recently Bim mRNA has also been identified as a target of the potentially oncogenic miR-32 and miR-17-92 microRNA clusters [44],[45],[46],[47].

Although it has been appreciated for several years that latent infection with EBV can produce a dramatic reduction in the amount of Bim expressed in B cells, at the onset of this study it remained unclear at precisely what level and by what molecular mechanism EBV regulates Bim expression. By demonstrating that EBV infection leads to a significant down-regulation of Bim mRNA levels and that the reduction in Bim protein does not appear to depend on ERK signalling [4], proteasome function or changes in Bim mRNA turnover (this study), we have focused attention on *Bim* transcription as the primary target of EBV. Consistent with these observations, EBV-mediated repression of *Bim* was shown to be associated with reduced acetylation on histones H3 and H4. Moreover Bim expression in EBV-infected cells was sensitive to agents that inhibit the maintenance of epigenetic marks on the histone and DNA components of chromatin. Taken together these results indicate that EBV primarily regulates the expression of Bim by reducing the rate of transcription through epigenetic mechanisms that may be initiated and/or maintained by the functional interaction of EBNA3A and EBNA3C [4].

Epigenetic modifications change chromatin structure without altering DNA sequence and they modify gene expression in a heritable manner. There are two closely linked components of the epigenome – methylation of cytosine in CpG dinucleotides and covalent modifications to the N-terminal tails of histones that alter chromatin organisation and function. Combined, these modifiers of gene expression can facilitate the long-term repression or silencing of genes [28],[34]. Changes in epigenetic marks – particularly regional gains in CpG methylation associated with the silencing of tumour suppressor genes – are common in most cancers [27]. Recently there has been speculation that infectious agents may induce epigenetic modifications that could contribute to the development of human tumours or other chronic conditions ([48] and see below). It is generally concluded that epigenetic repression is progressive and is probably initiated by preventing activation and removing the acetylation marks on histones that ensure chromatin has an ‘open’ active configuration. This is followed by covalent attachment of repressive marks on histones and subsequently promoter DNA methylation [28],[34],[35]. In the B cells we have investigated here, it is probable that EBV has inhibited the transcription of Bim – via a mechanism involving EBNA3A and 3C and repressive marks on local chromatin – and prevented the efficient assembly of transcription complexes at or around the transcription initiation site. This then made the unusually large CpG-island available for DNA methylation. The demonstration that DNA methylation is probably a relatively late event after infection with EBV prompted us to investigate a histone modification associated with transcriptional repression. Trimethylation on lysine 27 of histone H3 has been shown in cancer cells to be a precursor to CpG methylation on DNA ([38],[39],[40],[41], reviewed in [42]). H3K27-Me3 of *Bim* occurs in low passage LCLs and *in vitro* ‘converts’, and this modification then creates an ideal substrate for the natural selection of the more stable epigenetic mark DNA methylation. These data provide compelling support for a model for EBV-associated lymphomagenesis that involves virus induced epigenetic reprogramming.

Currently we do not know precisely where or how H3K27-Me and CpG methylation are ‘seeded’ or the rate or mechanism of spread or even whether we have focused on the functionally important nucleotides in the CpG island. Nor do we understand the roles of EBNA3A and EBNA3C in the process. Nevertheless, since presently the only known cellular histone methyltransferase (HMT) capable of methylating H3K27 is a polycomb group (PcG) protein called EZH2 [42] then it is tempting to speculate that EBV may be utilizing the PcG family of chromatin regulators to suppress expression of *Bim*. Whatever the mechanisms, we suggest that spread of DNA methylation and stable repression would be driven, at least in BL, by the strong selection pressure to prevent Bim-mediated apoptosis induced by deregulated Myc [10],[11].

In summary, we propose that when EBV infects naive B cells and type III latency is established, that *Bim* is down-regulated through the actions of EBNA3A and EBNA3C and that this is necessary because EBNA2 activates *Myc*, which would otherwise induce an increased amount of Bim and apoptosis (see [4],[49]). This involves epigenetic changes on the *Bim* promoter that may prevent its activation by deregulated *Myc* and reduces the expression of Bim below a critical threshold. Thus *Myc* activation by EBNA2 – or a translocation event as is found in all BL – can be tolerated in an EBV-infected B cell. The initial epigenetic marks on *Bim* make the CpG-island available for DNA methylation capable of reinforcing the inhibition of transcription. By definition epigenetic changes will be inherited by progeny cells and during the pathogenesis of EBV-positive BL there will be continuous selection pressure to ensure that the suppression of *Bim* is retained. Moreover EZH2, the histone methyltransferase responsible for the H3K27-Me3 mark, can specifically recruit DNA methyltransferases (DNMTs) and so accelerate local CpG methylation [38]. This will continue to be the case after the proteins that initiated the reduction of transcription are no longer expressed in the EBNA1-only type I latency characteristic of BL [3].

To our knowledge EBV encodes no DNMTs but we cannot rule out interactions between EBV factors and cellular DNMTs (similar to those reported for KSHV LANA1 protein and the papillomavirus nuclear oncoproteins) being involved in the targeted repression of *Bim* (see [50],[51]). The recent discovery that KSHV, via the LANA1 protein, can down-regulate expression of the TGFβ-type II receptor and initiate methylation of its promoter [52], suggests that epigenetic modification of the host genome by viruses capable of a latent infection could be a common feature during disease pathogenesis. At this stage direct interactions between EBV factors and PcG proteins should not be excluded.

Finally it should be noted that screens for promoter methylation in a variety of other cancers (including breast and ovarian) have revealed no significant or consistent methylation of the *Bim* promoter using the same MSP primer sets as those used in this study (PS – unpublished data). Furthermore we have seen no consistent down-regulation of any other BH3-only pro-apoptotic proteins (including Bid, Noxa or Puma) in EBV-positive relative to EBV-negative B cells (our unpublished data). It is interesting to note that CpG methylation of *Puma* was recently demonstrated in Myc-driven B cell lymphomas – including some BL – but this was not dependent on infection with EBV and may even be absent in cells expressing the latency III pattern of EBV genes [53].

## Supporting Information

Figure S1 Degradation of Myc and β-Actin mRNAs following treatment with actinomycin D. A similar experiment to that shown in Figure 1B was done to test mRNA degradation rates of a rapidly degraded mRNA–Myc–and a slower degraded mRNA–β-Actin, as controls. In the top graph the rate of Myc mRNA degradation is shown. The rate of degradation appears greater for Myc mRNA, relative to Bim mRNA, as expected. In the second graph the rate of degradation of β-Actin mRNA is shown. This is slower than both Myc and Bim mRNA, as expected. After the first hour of treatment with Act D, the levels of β-Actin mRNA seems to go down faster in uninfected cells, relative to infected ones. Because a constant amount of RNA was used to assess Bim mRNA levels at each time point, this is probably due to uninfected cells dying faster than infected ones, and faster than the normal turnover of β-Actin mRNA. Hence, the levels of β-Actin mRNA follow the rate of cell death, in this case. The relative amount of mRNAs in these cells at the start of the treatment is shown in the bottom graph.
(1.65 MB TIF)

Figure S2 Inhibitors of HDACs and DNMTs up-regulate Bim protein levels in EBV infected cells (without inducing the lytic cycle) in *in vitro* converts and latency I cells. A) EBV-negative BL lines BL41 and Ramos and EBV-positive BL41 and Ramos AW were treated with TSA, AZA and Na But. Bim protein levels were assessed by western immunoblotting. In EBV-positive cells there is a slight increase of Bim levels, especially after treatment with TSA and Na But as in Figure 3. B) Latency I Eli and Akata 6 cells were treated with TSA and AZA. Bim protein levels increase slightly, especially after AZA treatment. C) To test whether treatment with these drugs induced the EBV lytic cycle, and in this way possibly affect *Bim* expression, the level of transcriptional activator of lytic genes BZLF1 was assessed by western immunoblotting. BZLF1 was not present at higher levels in BL31 cells infected with EBV (BL31 WT) or LCL CH cells after treatment with these inhibitors. This suggests that the lytic cycle was not induced by the treatments. BL31 E2KO cells have increased BZLF1 levels after TSA treatment and they were used as positive control. D) To verify that cell lines considered to have the latency I expression pattern had not ‘drifted’ to latency III, western immunoblots were performed to assess EBNA3A and EBNA3C expression. The EBV-negative cell line BL31 and latency I Eli, Akata 6 and Dante cell lines did not have detectable levels of either protein, indicating that these latency I cell lines had not significantly ‘drifted’ to latency III.
(2.77 MB TIF)

Figure S3 MSP analysis of cell lines. Amplified DNA by MSP was visualized by agarose gel electrophoresis, for all primer sets (I–V), with primer pairs specific for unmethylated state (U) or methylated state (M). Here, as a representative example, the results for cell lines with primer set III are shown to demonstrate how these were interpreted to produce the matrix in Figure 5. DNA from primary B cells was used as a negative control for DNA methylation and *in vitro* methylated Jurkat DNA (Meth) as a positive control.
(1.76 MB TIF)

Figure S4 MSP analysis of DNA from biopsy samples. To demonstrate how MSP data were produced for presentation in the matrix in Figure 7, DNA amplified by MSP using each set of primers (I–V) and visualized by agarose gel electrophoresis is shown. For each primer set, primer pairs specific for unmethylated state (U) or methylated state (M) were used. DNA from primary B cells was used as a negative control for DNA methylation and *in vitro* methylated Jurkat DNA (Meth) as a positive control.
(2.40 MB TIF)

Figure S5 Controls for methylated DNA precipitations. Precipitations were performed as described in Figure 10A using DNA extracted from PBMCs (as a control for unmethylated DNA and DNA (Meth) from Jurkat cells methylated *in vitro* [(Meth) as a control for fully methylated DNA]. There is no DNA methylation at the *Bim* promoter of PBMCs and no enrichment was observed. For *in vitro* fully methylated DNA, the enrichment observed was determined by the concentration of CpG dinucleotides at the locus assessed by the particular primer pair. For primer pair A, which is outside the CpG-island, there was no significant enrichment. There was also no DNA precipitated when only the magnetic beads were used, without the His-MBD2b protein attached.
(1.53 MB TIF)
