# Supplementary figures and images for: Epstein-Barr Virus Latency in B Cells Leads to Epigenetic Repression and CpG Methylation of the Tumour Suppressor Gene Bim
Source: PLoS Pathog. 2009 Jun 26;5(6):e1000492. doi: 10.1371/journal.ppat.1000492 (PMC2695769; doi:10.1371/journal.ppat.1000492)

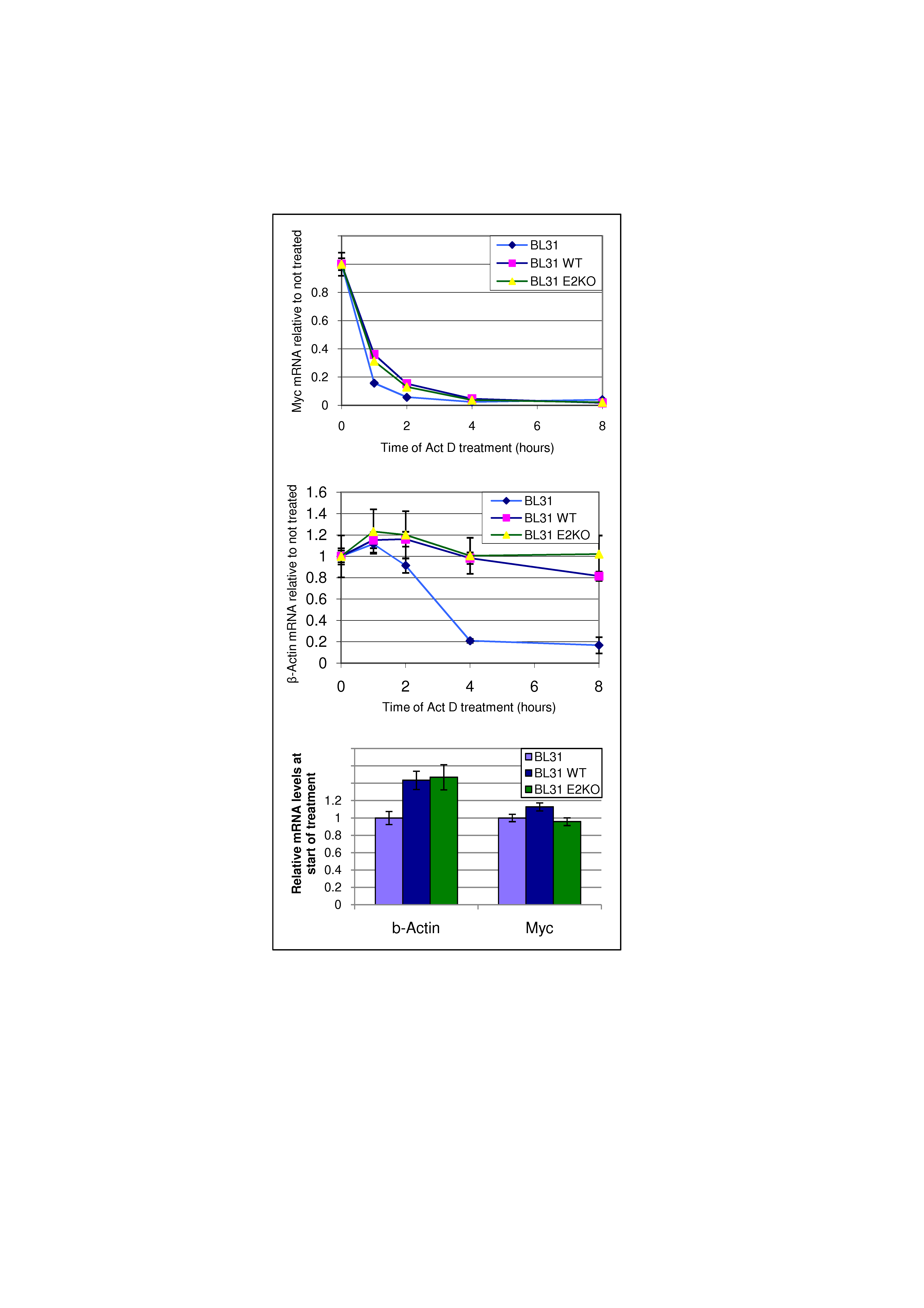

Supplement: Figure S1 — Degradation of Myc and β-Actin mRNAs following treatment with actinomycin D. A similar experiment to that shown in Figure 1B was done to test mRNA degradation rates of a rapidly degraded mRNA–Myc–and a slower degraded mRNA–β-Actin, as controls. In the top graph the rate of Myc mRNA degradation is shown. The rate of degradation appears greater for Myc mRNA, relative to Bim mRNA, as expected. In the second graph the rate of degradation of β-Actin mRNA is shown. This is slower than both Myc and Bim mRNA, as expected. After the first hour of treatment with Act D, the levels of β-Actin mRNA seems to go down faster in uninfected cells, relative to infected ones. Because a constant amount of RNA was used to assess Bim mRNA levels at each time point, this is probably due to uninfected cells dying faster than infected ones, and faster than the normal turnover of β-Actin mRNA. Hence, the levels of β-Actin mRNA follow the rate of cell death, in this case. The relative amount of mRNAs in these cells at the start of the treatment is shown in the bottom graph. (1.65 MB TIF) [file ppat.1000492.s001.tif]

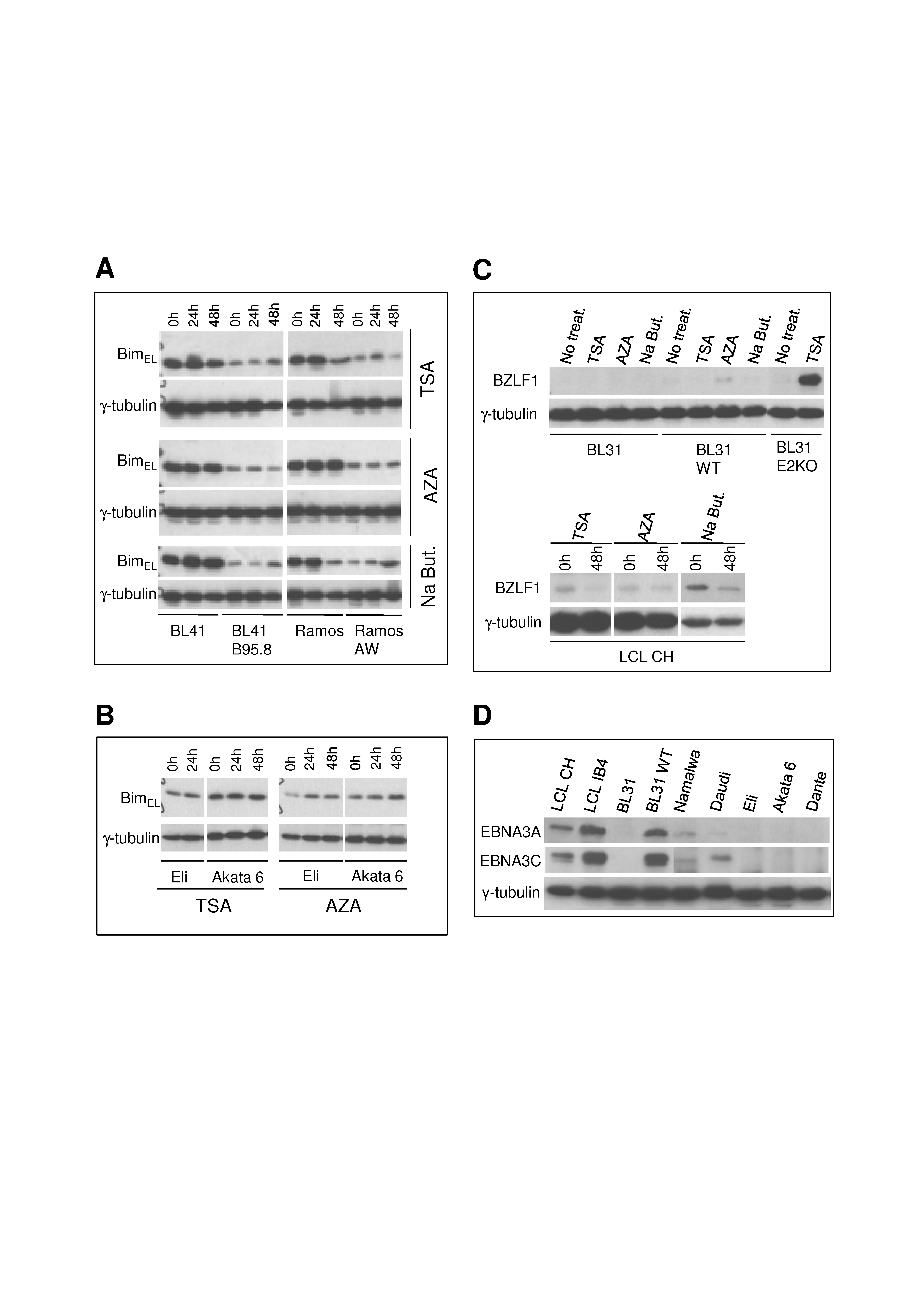

Supplement: Figure S2 — Inhibitors of HDACs and DNMTs up-regulate Bim protein levels in EBV infected cells (without inducing the lytic cycle) in in vitro converts and latency I cells. A) EBV-negative BL lines BL41 and Ramos and EBV-positive BL41 and Ramos AW were treated with TSA, AZA and Na But. Bim protein levels were assessed by western immunoblotting. In EBV-positive cells there is a slight increase of Bim levels, especially after treatment with TSA and Na But as in Figure 3. B) Latency I Eli and Akata 6 cells were treated with TSA and AZA. Bim protein levels increase slightly, especially after AZA treatment. C) To test whether treatment with these drugs induced the EBV lytic cycle, and in this way possibly affect Bim expression, the level of transcriptional activator of lytic genes BZLF1 was assessed by western immunoblotting. BZLF1 was not present at higher levels in BL31 cells infected with EBV (BL31 WT) or LCL CH cells after treatment with these inhibitors. This suggests that the lytic cycle was not induced by the treatments. BL31 E2KO cells have increased BZLF1 levels after TSA treatment and they were used as positive control. D) To verify that cell lines considered to have the latency I expression pattern had not ‘drifted’ to latency III, western immunoblots were performed to assess EBNA3A and EBNA3C expression. The EBV-negative cell line BL31 and latency I Eli, Akata 6 and Dante cell lines did not have detectable levels of either protein, indicating that these latency I cell lines had not significantly ‘drifted’ to latency III. (2.77 MB TIF) [file ppat.1000492.s002.tif]

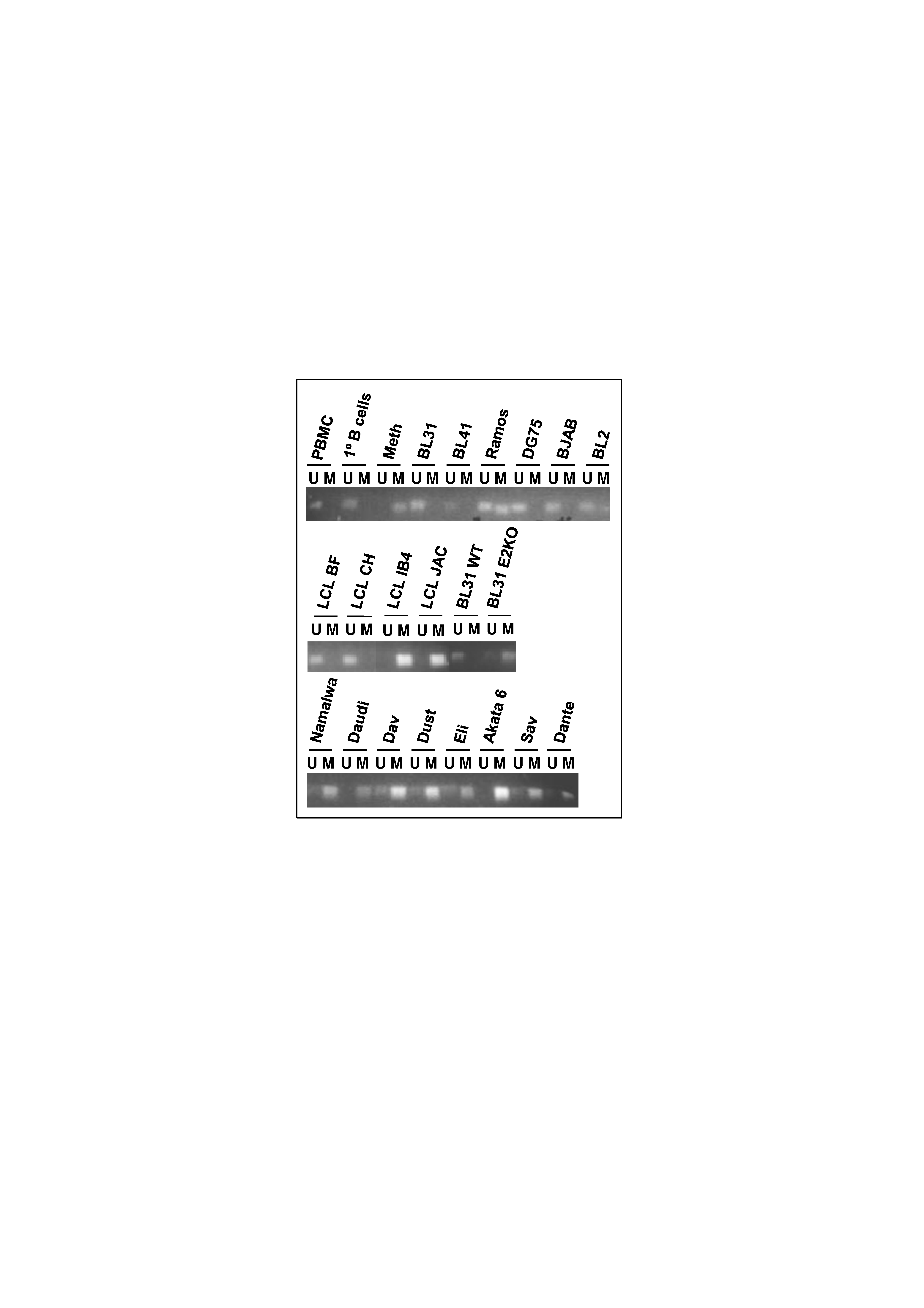

Supplement: Figure S3 — MSP analysis of cell lines. Amplified DNA by MSP was visualized by agarose gel electrophoresis, for all primer sets (I–V), with primer pairs specific for unmethylated state (U) or methylated state (M). Here, as a representative example, the results for cell lines with primer set III are shown to demonstrate how these were interpreted to produce the matrix in Figure 5. DNA from primary B cells was used as a negative control for DNA methylation and in vitro methylated Jurkat DNA (Meth) as a positive control. (1.76 MB TIF) [file ppat.1000492.s003.tif]

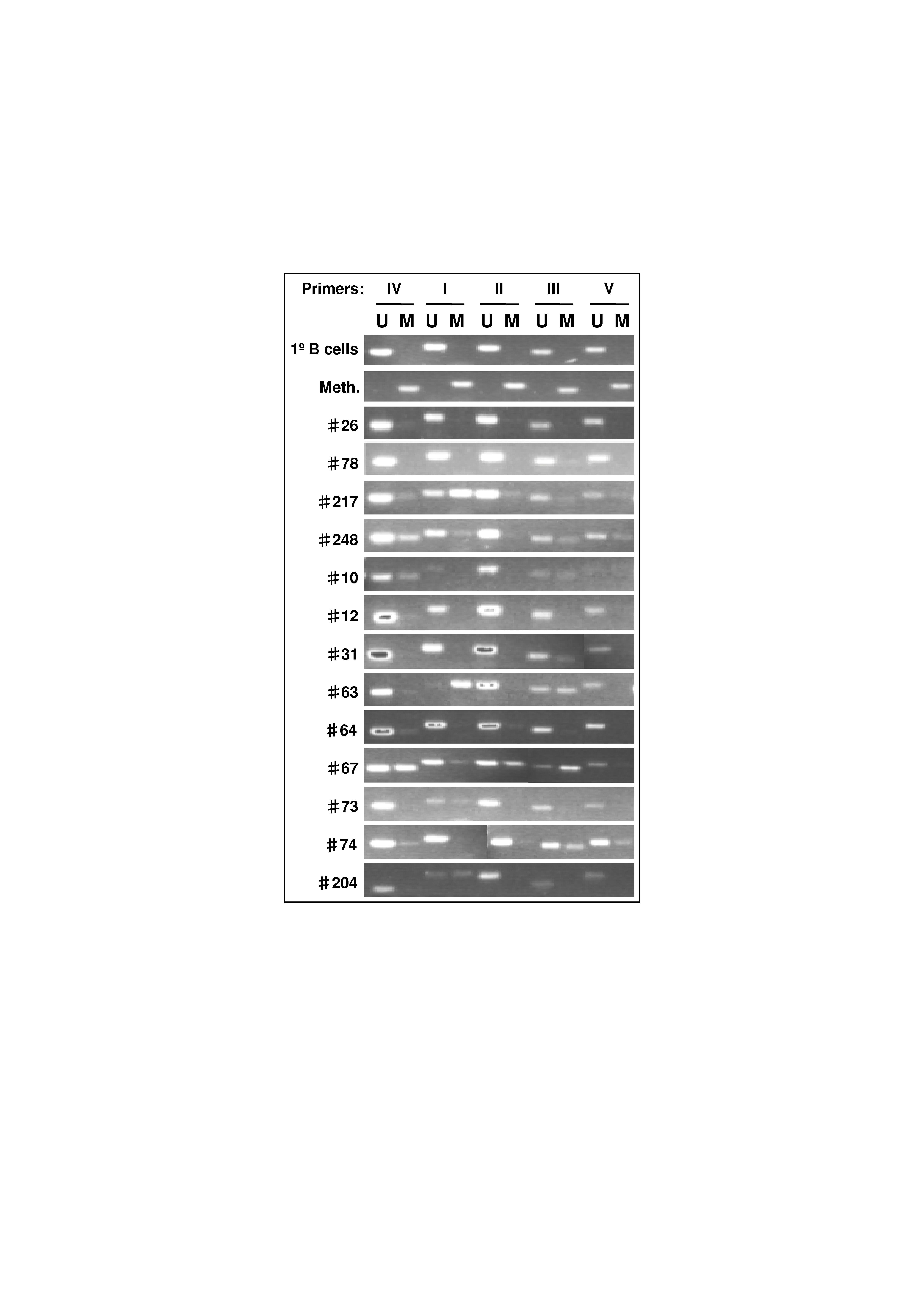

Supplement: Figure S4 — MSP analysis of DNA from biopsy samples. To demonstrate how MSP data were produced for presentation in the matrix in Figure 7, DNA amplified by MSP using each set of primers (I–V) and visualized by agarose gel electrophoresis is shown. For each primer set, primer pairs specific for unmethylated state (U) or methylated state (M) were used. DNA from primary B cells was used as a negative control for DNA methylation and in vitro methylated Jurkat DNA (Meth) as a positive control. (2.40 MB TIF) [file ppat.1000492.s004.tif]

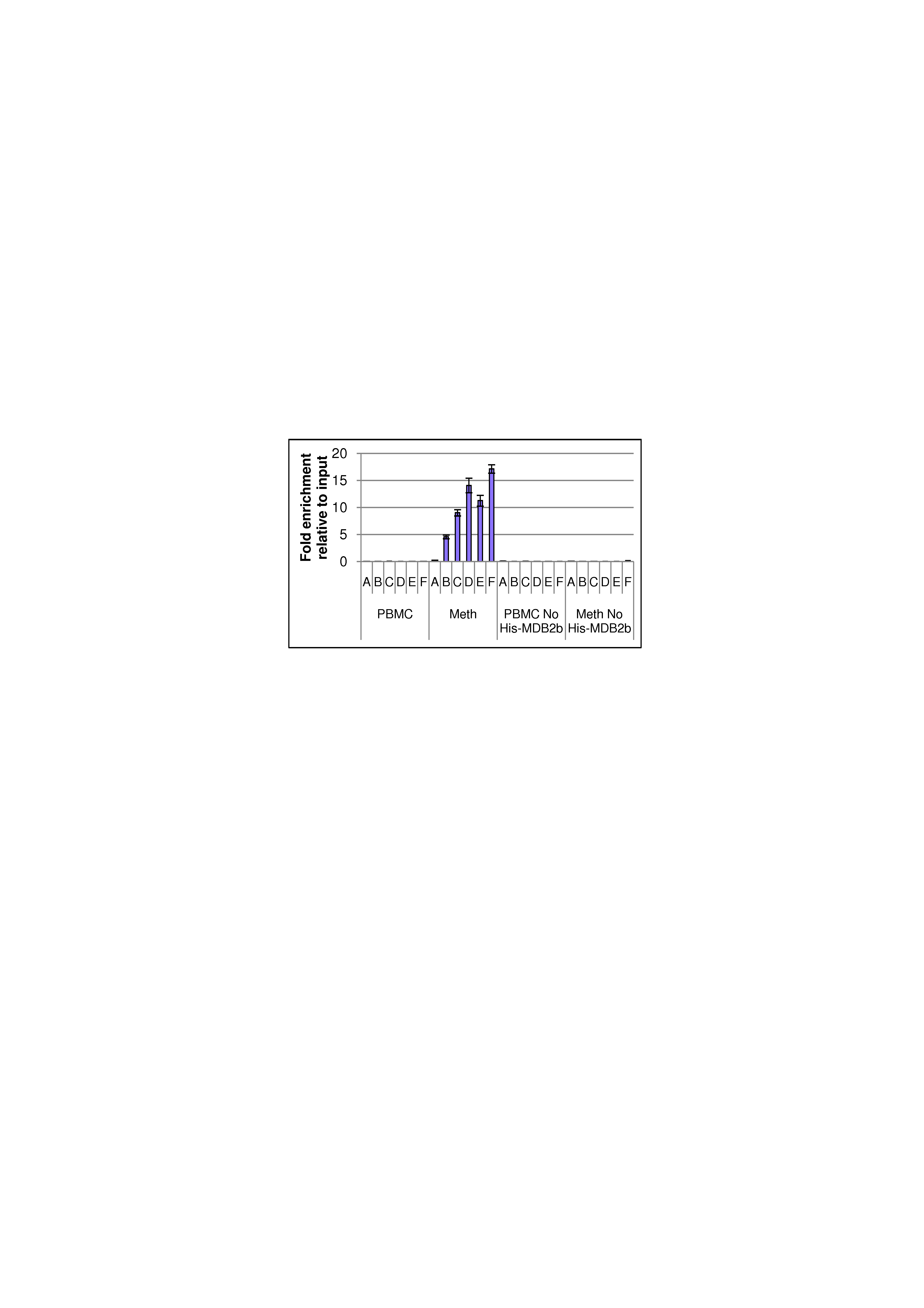

Supplement: Figure S5 — Controls for methylated DNA precipitations. Precipitations were performed as described in Figure 10A using DNA extracted from PBMCs (as a control for unmethylated DNA and DNA (Meth) from Jurkat cells methylated in vitro [(Meth) as a control for fully methylated DNA]. There is no DNA methylation at the Bim promoter of PBMCs and no enrichment was observed. For in vitro fully methylated DNA, the enrichment observed was determined by the concentration of CpG dinucleotides at the locus assessed by the particular primer pair. For primer pair A, which is outside the CpG-island, there was no significant enrichment. There was also no DNA precipitated when only the magnetic beads were used, without the His-MBD2b protein attached. (1.53 MB TIF) [file ppat.1000492.s005.tif]
